# Pulmonary hypertension complicating pulmonary sarcoidosis

**DOI:** 10.1007/s12471-016-0847-1

**Published:** 2016-05-18

**Authors:** M. P. Huitema, J. C. Grutters, B. J. W. M. Rensing, H. J. Reesink, M. C. Post

**Affiliations:** 1 Department of Cardiology, St. Antonius Hospital Nieuwegein, Nieuwegein, The Netherlands; 2 Department of Pulmonology, St. Antonius Hospital Nieuwegein, Nieuwegein, The Netherlands; 3 Division of Heart and Lung, UMC Utrecht, Utrecht, The Netherlands

**Keywords:** Pulmonary hypertension, Sarcoidosis, Interstitial lung disease

## Abstract

Pulmonary hypertension (PH) is a severe complication of sarcoidosis, with an unknown prevalence. The aetiology is multifactorial, and the exact mechanism of PH in the individual patient is often difficult to establish. The diagnostic work-up and treatment of PH in sarcoidosis is complex, and should therefore be determined by a multidisciplinary expert team in a specialised centre. It is still a major challenge to identify sarcoidosis patients at risk for developing PH. There is no validated algorithm when to refer a patient suspected for PH, and PH analysis itself is difficult. Until present, there is no established therapy for PH in sarcoidosis. Besides optimal treatment for sarcoidosis, case series evaluating new therapeutic options involving PH-targeted therapy are arising for a subgroup of patients. This review summarises the current knowledge regarding the aetiology, diagnosis and possible treatment options for PH in sarcoidosis.

## Introduction

Sarcoidosis is a rare, multisystemic disorder of unknown aetiology. It is characterised by non-caseating granulomas which present in multiple tissues, particularly in the lung and lymphatic system. The course of sarcoidosis can vary from spontaneous resolution to severe and chronic disease. It is often complicated by pulmonary fibrosis [1].

Pulmonary hypertension (PH) is defined as a mean pulmonary artery pressure (PAP) of ≥25 mmHg at rest measured by right heart catheterisation [2]. PH is increasingly recognised as a serious complication of pulmonary sarcoidosis. The clinical diagnosis or suspicion of PH in sarcoidosis is challenging, since symptoms overlap. PH exists in all stages of sarcoidosis; however, the frequency largely depends on the severity of the sarcoidosis [3, 4]. There is a lack of robust data on accurate diagnostic tools and therapeutic options for PH in sarcoidosis. However, diagnostic tests are in further development, and case series of potential treatment options are arising. Diagnosis and treatment decisions should be made in a specialised team, including a dedicated cardiologist, pulmonologist, radiologist and nurse. In this review, we will discuss the classification and aetiology of sarcoidosis-associated PH (SAPH), diagnostic tests and possible treatment options.

## Epidemiology

There are no clear data regarding the true prevalence of SAPH. Rates of PH in sarcoidosis patients in tertiary centres vary between 5–20 % [3, 5, 6]. As a side note, the gold standard right heart catheterisation for diagnosing PH is often absent in studies, where results are based on echocardiography only. The prevalence of PH may increase to > 50 % in sarcoidosis patients with persistent or unexplained dyspnoea [4, 7]. A PH prevalence of more than 70 % was found in sarcoidosis patients awaiting lung transplant [8, 9].

## Prognosis

Mortality in sarcoidosis patients is significantly increased if PH is present, independent of pulmonary function [10, 11]. For 22 patients with haemodynamically confirmed SAPH, the 1-, 2‑, and 5‑year survival was found to be 84, 74 and 59 %, compared with 100, 96 and 96 % in matched sarcoidosis patients without PH (*p* = 0.003) [10]. Another, non-invasive study showed an echocardiographic estimation of systolic PAP >50 mmHg to be associated with increased mortality [7].

## Classification of PH in sarcoidosis

According to the World Health Organisation, PH is classified into five different groups, based on clinical presentation, pathological findings, haemodynamic characteristics and treatment strategy (Tab. 1; [2]). SAPH is classified in group 5: PH with an unknown and multifactorial mechanism. However, PH in sarcoidosis can be classified in group 2, 3 or 4 as well, if the underlying mechanism is clear. For example, a patient with severe fibrotic abnormalities with a mean PAP that is in line with the severity of the lung disease will be classified as group 3 PH. On the other hand, if a patient presents with mild lung disease, but the mean PAP is high (e. g. >35 mmHg), a component of vasculopathy can be suspected. The haemodynamic profile might show similarities with pulmonary arterial hypertension. Because of the unknown mechanism, this patient will be classified as group 5 PH.

**Tab. 1:** Clinical classification of pulmonary hypertension [2]

WHO group 1	Pulmonary arterial hypertension	
WHO group 2	Pulmonary hypertension due to left heart disease	
WHO group 3	Pulmonary hypertension due to lung disease and/or hypoxaemia	
WHO group 4	Chronic thromboembolic pulmonary hypertension or other pulmonary artery obstructions	
WHO group 5	Pulmonary hypertension with unclear multifactorial mechanism	
	5.1	Hematologic disorders: myeloproliferative disorders, splenectomy, chronic haemolytic disorders
	5.2	Systemic disorders: sarcoidosis, pulmonary Langerhans cell histiocytosis lymphangioleiomyomatisos
	5.3	Metabolic disorders: glycogen storage disease, Gaucher disease, thyroid disorders
	5.4	Others: pulmonary tumoral thrombotic microangiopathy, fibrosing mediastinitis, chronic renal failure, segmental pulmonary hypertension

*WHO* World Health Organisation

## Aetiology of PH in sarcoidosis

The aetiology of PH in sarcoidosis is multifactorial. Based on the pulmonary artery wedge pressure (PAWP), SAPH can be divided into pre-capillary (PAWP ≤15 mmHg) and post-capillary (PAWP >15 mmHg) PH [2].

### Pre-capillary PH

Pre-capillary PH in sarcoidosis might be due to one or several mechanisms. Hypothesised mechanisms are described below.

#### Destruction of distal capillary bed and resultant hypoxaemia

Hypoxic vasoconstriction due to destruction of the distal capillary bed is one of the first possible mechanisms of SAPH. Data to support this mechanism are scarce, ranging from no difference in oxygen saturation or need for supplemental oxygen between sarcoidosis patients with and without PH [4], to the need of supplemental oxygen as only predictor of SAPH in patients listed for lung transplantation [8]. The majority of patients with SAPH suffer a more advanced stage of sarcoidosis, often in the presence of pulmonary fibrosis [3, 5, 7, 10]. However, approximately 32–50 % of the patients with SAPH have no significant fibrosis [3, 5, 10], suggesting that fibrotic ablation of the pulmonary vasculature is not the only mechanism to explain SAPH.

#### Specific vasculopathy

Involvement of granulomatous vasculopathy is another possible mechanism of SAPH [12]. One study reported vascular involvement in all 40 autopsies of patients with mainly pulmonary and cardiac sarcoidosis [13]. Granulomatous vasculopathy causes frequent occlusion of arterioles or venules [13] and angiitis [14], which might result in PH. Sarcoid granulomas can be found throughout all layers of the pulmonary vascular wall, often in combination with intimal fibrosis, medial proliferation, inflammatory changes, necrosis and destruction of the small-vessel architecture, which leads to an occlusive vasculopathy [10, 13]. Granulomas can also be formed at the level of the intima in large vessels. These patients are often misclassified as having chronic thromboembolic pulmonary hypertension.

#### Local increased vasoreactivity

Acute vasoresponsiveness is defined as a decrease in mean PAP of ≥10 mmHg to an absolute value of mean PAP ≤40 mmHg, with an increased or unchanged cardiac output [2]. Several patients with SAPH have shown responsiveness to acute vasodilator challenge with inhaled nitrogen oxide or prostacyclin. Preston et al. [15] showed a decrease of 18 ± 4 % in mean PAP and 31 ± 5 % in pulmonary vascular resistance in seven of eight patients receiving inhaled nitric oxide, with a slight increase of cardiac output. For epoprostenol, no significant change was reported. Fisher et al. [16] showed an average decrease in pulmonary vascular resistance of 45 %, and a non-significant decrease in mean PAP of 11 % in six out of seven patients using epoprostenol. However, an increased vasoreactivity is not a predictor for response to PH-targeted therapy in pulmonary arterial hypertension, but highlights the potential role of an increased reactivity of the pulmonary vasculature as part of the unclear pathogenesis of PH in sarcoidosis.

#### Extrinsic compression of pulmonary vessels

**Fig. 1:**
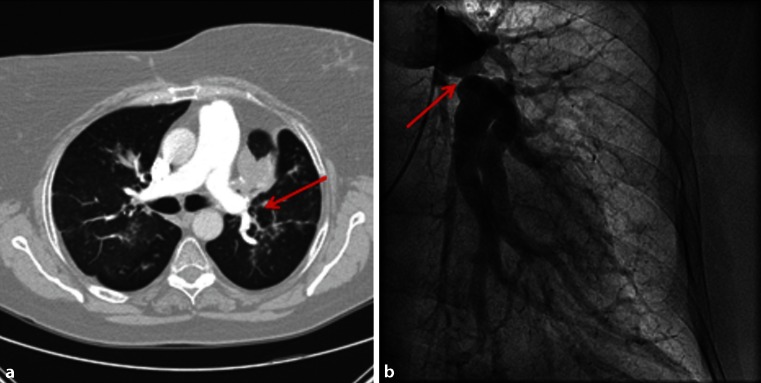
Compression of the pulmonary artery. A case of a 60-year-old female with stage IV sarcoidosis, diagnosed with PH with a mean PAP of 63 mmHg, due to compression of the pulmonary artery. **a** Compression on chest CT; **b** Pulmonary angiogram of the left lobe

Extrinsic compression of the pulmonary arteries, and less frequently the pulmonary veins, by enlarged lymph nodes or fibrosing mediastinitis, has also been described as a mechanism for SAPH [10, 13, 17]. Fig. 1 shows an example of a sarcoidosis patient with PH due to compression of the pulmonary artery. Compression of the pulmonary artery occurs more frequently in chronic sarcoidosis, and presented in 21 % of the PH patients with radiographic stage IV sarcoidosis [10].

#### Portal hypertension

Hepatic sarcoidosis is present in up to 70 % of sarcoidosis patients, and might lead to PH due to cirrhosis and portal hypertension [18]. Approximately 1–5 % of patients with portal hypertension develop PH [2], with liver cirrhosis being the most common cause of portal hypertension.

#### Pulmonary veno-occlusive disease

Pulmonary veno-occlusive disease is defined as extensive narrowing or occlusion of the pulmonary veins in lobular septa or intra-acinar venules by fibrous tissue [19]. In a case series of five patients with SAPH who underwent lung transplant, all explanted lungs demonstrated presence of pulmonary veno-occlusive disease [10].

### Post-capillary PH

Apart from pre-capillary PH, post-capillary PH can also be found in sarcoidosis. Systolic, diastolic and subclinical left ventricular dysfunction are often present in sarcoidosis [20]. In a retrospective study consisting of 130 sarcoidosis patients of all stages, with persistent moderate to severe dyspnoea, who underwent right heart catheterisation after at least six months of immunosuppressive therapy, the prevalence of PH was 54 %. Interestingly, in 29 % an elevated PAWP >15 mmHg was measured, suggesting left ventricular disease as a cause of PH [7]. Left ventricular dysfunction might be due to myocardial involvement of sarcoidosis. Autopsy studies suggest that at least 25 % of the patients with sarcoidosis have myocardial involvement. The diagnosis of cardiac sarcoidosis is difficult, because signs and symptoms can be subtle or even absent. The expert consensus statement advises to screen for cardiac sarcoidosis using cardiac magnetic resonance imaging (CMR) and 18 F-fluorodeoxyglucose positron emission tomography in sarcoidosis patients at risk for myocardial involvement [21].

## Diagnosis

Establishing the diagnosis of PH in sarcoidosis is challenging. Symptoms suggestive for PH are dyspnoea, dizziness, cough and chest pain, or signs such as elevated jugular venous pressure, S3 or S4 heart sounds, lower extremity oedema or right ventricular heave. These signs are usually associated with more advanced PH [4]. During follow-up, several tests may raise suspicion of PH, requiring PH analysis.

### Suspicion of PH

#### Pulmonary function test

Several parameters of pulmonary function testing are associated with SAPH, such as a decreased forced vital capacity, total lung capacity [5] and decreased diffusion capacity of carbon monoxide [3]. However, PH also occurs in sarcoidosis patients with near-normal lung function tests. An echocardiographic study reports a prevalence of PH of 28 % in 127 sarcoidosis patients with near-normal lung function tests, defined as a forced vital capacity >70 %, forced expiratory volume in 1 second >70 %, and diffusion capacity of carbon monoxide >60 % [22]. Therefore, PH is suspected in the presence of progressive dyspnoea, while pulmonary function tests remain stable.

#### Exercise testing

The 6‑minute walk test (6MWT) can also raise suspicion for PH. Most sarcoidosis patients with PH will have a 6MWT distance of less than 450 meters [3, 23–27]. An oxygen desaturation to a value below 90 % during the 6MWT is associated with PH [3]. However, a major limitation of the 6MWT is that results may be influenced by other factors, such as airway disease, cardiac disease, fatigue and muscle involvement [27].

Cardiopulmonary exercise testing for detecting PH has not been studied systematically in sarcoidosis. In patients with pulmonary arterial hypertension, reduced maximum oxygen uptake (VO2max), increased ventilator inefficiency, early lactate acidosis, decreased end-expiratory CO2 and reduced O2 pulse are an indication for pulmonary arterial hypertension, although these parameters are non-specific [28]. One study evaluated exercise testing in patients with interstitial lung disease. Peak exercise capacity was significantly lower in interstitial lung disease patients with PH compared with those without PH. Also, carbon dioxide production and load (Watts) was lower, and the ventilation/carbon dioxide production ratio at ventilatory threshold was higher in patients with PH [29].

#### Chest computed tomography

**Fig. 2:**
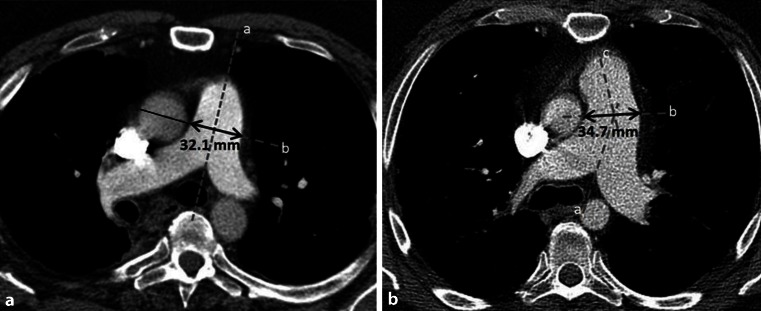
Measurement of the pulmonary artery on chest CT. **a** Measurement of the main pulmonary artery, at the level of the bifurcation along the line that originates from the centre of the adjacent ascending aorta, perpendicular to the axis of main pulmonary artery; **b** If the trunk is too curved, measurements can be made using the other method [32]

A dilated pulmonary artery on chest computed tomography (CT) may raise suspicion of PH [2]. The cut-off value is ≥29 mm for the pulmonary artery, or a ratio of the pulmonary artery diameter to the ascending aorta diameter of ≥1 [2]. Fig. 2 shows the measurement of the pumonary artery diameter on chest CT. Two studies [30, 31] found no correlation between the pulmonary artery diameter and the mean PAP in patients with fibrotic lung disease. However, a recently published article investigating only sarcoidosis patients [32], showed a good correlation for the pulmonary artery diameter indexed for body surface area and the presence of PH; this had a high diagnostic accuracy for discriminating between the presence and absence of PH. Other chest CT parameters are right ventricular enlargement, or a segmental artery:bronchus ratio > 1:1. However, they seem to have less value in clinical practice.

#### Cardiac magnetic resonance imaging

CMR is often used for detecting cardiac sarcoidosis. In case of suspicion of PH, CMR is a valuable tool to accurately and reproducibly assess the right ventricular dimension, morphology and function, stroke volume, cardiac output, pulmonary artery distensibility and right ventricular mass. Reduced pulmonary artery distensibility and retrograde flow are predictive for PH in patients at risk; however, this cannot exclude PH [33].

### Specific PH analysis

In general, the PH analysis protocol used in sarcoidosis patients is similar to the standard analysis protocol as described in the PH guideline [2]. The analysis consists of biomarkers, electrocardiogram (ECG) and echocardiography.

#### Biomarkers

The main biomarker for PH is brain natriuretic peptides (BNP). Recently, BNP has been studied in a right heart catheterisation study of a group of 113 patients with interstitial lung disease [34]. BNP was significantly increased in patients with an invasive systolic PAP >40 mmHg. However, BNP levels were significantly higher in patients with an increased PAWP. The correlation between BNP and the mean PAP was strong (r = 0.82, *p* < 0.001).

#### Electrocardiogram

Abnormalities on the ECG suggesting right ventricular overload often present in more severe PH. Therefore, ECG cannot be used to exclude the diagnosis. ECG abnormalities include P‑pulmonale, right axis deviation, right ventricular hypertrophy or strain, right bundle branch block and QTc prolongation [2].

#### Echocardiography

Echocardiography plays a key role in PH analysis [2]. The primary echocardiographic parameter for PH is the estimation of the systolic PAP based on the peak tricuspid regurgitation velocity (TRV) measured by continuous wave Doppler and the right atrial pressure [2]. Importantly, this measurement can overestimate or underestimate the actual pressures. A meta-analysis of 29 studies showed a modest diagnostic accuracy [35]. However, the accuracy drops if the tricuspid regurgitation jet is difficult to obtain, and if image quality is limited. Therefore, isolated use of the peak TRV is unreliable. Recently, the guidelines recommend using peak TRV in combination with additional parameters in order to divide patients according to the echocardiographic probability of PH [2]. Additional parameters include for example right ventricular dilatation and/or hypertrophy, decreased systolic right ventricular function by assessing tricuspid annulus plane systolic excursion and S‑wave on tissue Doppler imaging, pulmonary artery dilation, right atrial dilation, and pulmonary artery acceleration time [2]. The echocardiographic signs and subsequent classifications are summarised in Tab. 2 and 3.

**Tab. 2:** Echocardiographic probability of pulmonary hypertension in symptomatic patients with a suspicion of PH [2]

Echocardiographic pulmonary hypertension probability	Peak tricuspid regurgitation velocity (m/s)	Other signs^a^ for pulmonary hypertension on echocardiography
Low	≤2.8 or not measurable	No
Intermediate	≤2.8 or not measurable	Yes
	2.9–3.4	No
High	2.9–3.4	Yes
	>3.4	Not required

^a^Other signs are described in Tab. 3. To meet the criteria of other signs, at least two other signs of at least two different categories must be abnormal

**Tab. 3:** Echocardiographic signs suggestive for pulmonary hypertension [2]

The ventricles	Right ventricle/left ventricle basal diameter ratio >1.0	Flattening of the interventricular septum (left ventricular eccentricity index >1.1 in systole and/or diastole)	
Pulmonary artery	Pulmonary arterial acceleration time <105 msec and/or midsystolic notching	Early diastolic pulmonary regurgitation velocity >2.2 m/sec	PA diameter >25 mm
Inferior vena cava and right atrium	Inferior cava diameter >21 mm with decreased inspiratory collapse (<50 % with a sniff or <20 % with quiet inspiration)	Right atrial area (end-systole >18 cm^2^)	

*PA* pulmonary artery,* PH* pulmonary hypertension

The literature on echocardiographic findings in sarcoidosis patients is scarce, and the value of right ventricular systolic pressure and peak TRV is controversial. In patients with interstitial lung disease including pulmonary fibrosis, right ventricular systolic pressure was found to be less accurate and measurable in only 54 % of the patients [36, 37]. In an echocardiographic study of 80 sarcoidosis patients, TRV was measurable in 70 % of patients. There was a significant correlation (r = 0.62, *p* < 0.0001) between the non-invasive and invasive measurements. However, several patients could have been misclassified [7].

A benefit of echocardiography is the possibility to detect other cardiac abnormalities to explain dyspnoea. Besides PH, the right ventricular function is also associated with cardiac sarcoidosis and abnormal pulmonary function tests, and might even be an isolated finding [38].

#### Right heart catheterisation

Right heart catheterisation remains the gold standard for diagnosing PH [2]. The invasive nature of this diagnostic modality makes it unsuitable for routine use [39]. Right heart catheterisation is recommended in patients with an intermediate or high risk of PH, on echocardiography, with realistic treatment possibilities [2]. The mean PAP in patients awaiting lung transplantation was 9 mmHg higher in sarcoidosis patients compared with idiopathic pulmonary fibrosis, despite similar spirometric severity [40]. In patients with sarcoidosis, PAPs are often higher than expected by parenchymal involvement only [40]. In such cases, a mean PAP exceeding 35 mmHg has to be considered to be severe PH. Additional to interstitial lung disease, these patients are suspected for pulmonary vascular abnormalities [41].

## Recommendations for clinical practice

**Fig. 3:**
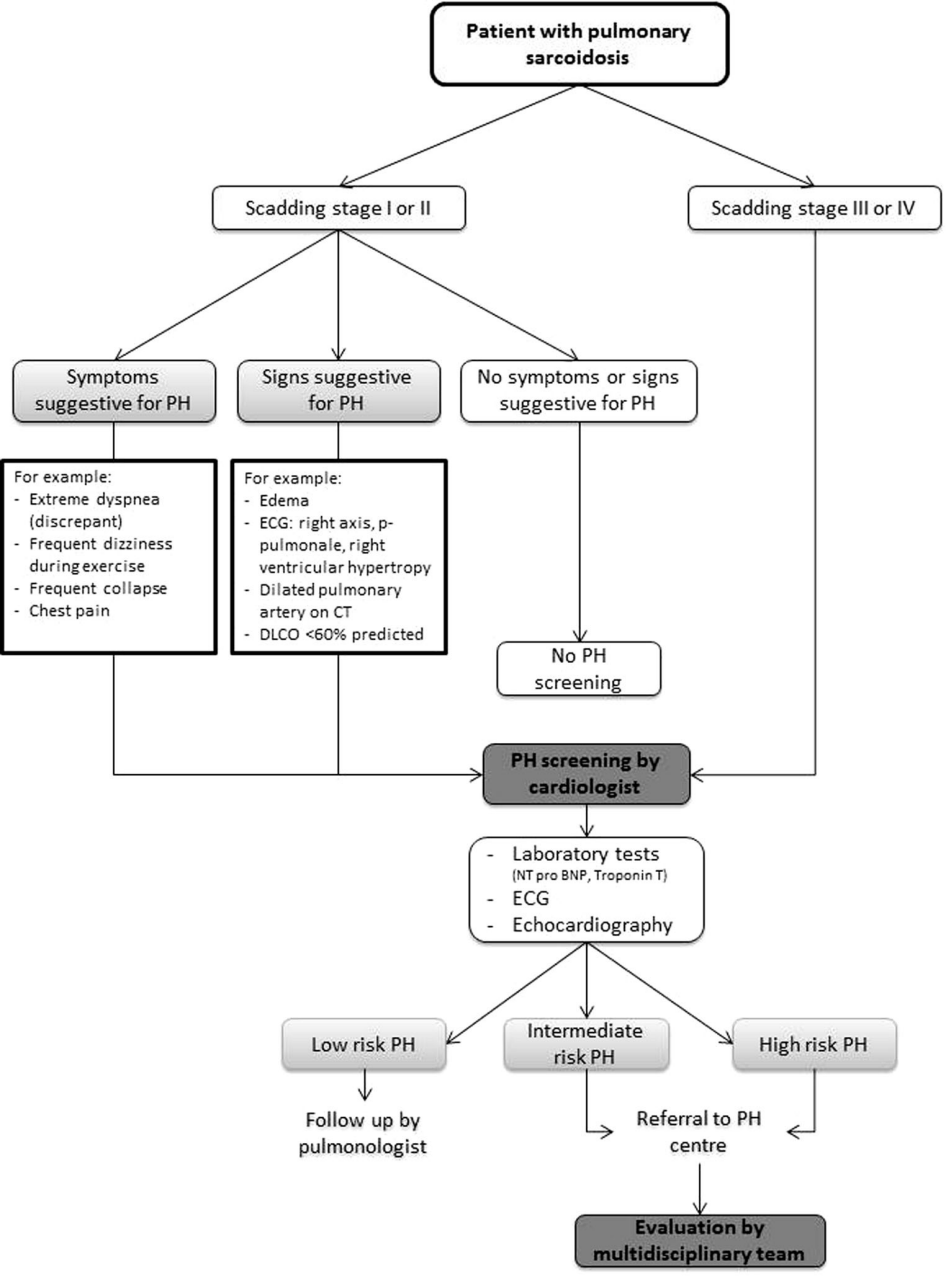
Flow chart for pulmonary hypertension screening in sarcoidosis

The literature regarding PH in sarcoidosis is scarce. Therefore, it is difficult to make evidence-based and clear recommendations as to which patients are at risk of developing PH, and on the best method for screening. Based on the current literature, we constructed a flow chart to give some guidance for screening (Fig. 3). Importantly, patients with intermediate to high risk for PH should be referred to a PH centre for further analysis.

## Management

In PH, a multidisciplinary approach involving cardiologists, pulmonologists and radiologists specialised in PH and interstitial lung disease is mandatory. Treatment of PH in sarcoidosis has only been studied in small groups, and there is no solid proof for the use of PH-targeted therapy in sarcoidosis. Therefore, treatment might benefit the individual patient, but there is no evidence for effectiveness. The treatment goal is to improve the vascular, haemodynamic and functional outcomes. Suggested therapies are targeted to the underlying mechanisms of PH in sarcoidosis. These strategies can be divided into sarcoidosis-targeted treatment and PH-targeted treatment. Both strategies will be described below.

### Sarcoidosis-targeted treatment

Sarcoidosis-targeted treatment might be indicated if the mechanism of PH is suspected to be due to sarcoidosis itself, for example in patients with compression of the pulmonary artery by lymphadenopathy. There is a step-wise approach for the management of sarcoidosis [42]. First-line treatment of sarcoidosis consists of oral glucocorticoids. However, long-term use on high doses is associated with substantial morbidity. As an alternative, antimetabolites such as methotrexate and azathioprine might be used. The next step would be anti-tumour necrosis factor monoclonal antibodies, or rituximab [42].

#### Anti-inflammatory and immunomodulatory agents

Anti-inflammatory and immunomodulatory agents potentially improve PH, if caused by active granulomatous inflammation. Nunes et al. [10] evaluated ten sarcoidosis patients (one with stage 0, four with stage II, and five with stage IV) after treatment with high doses of glucocorticosteroids. Patients with stage IV sarcoidosis did not respond, whereas in three patients (one with stage 0 and two with stage II) systolic PAP decreased by more than 20 % after 3–6 months of follow-up. Afterwards, these patients were maintained on low doses of corticosteroids, and the systolic PAP continued to decrease. This suggests that fibrotic disease is most likely not responsive to corticosteroids, whereas patients with active granulomatous inflammation might benefit. Patients with compression of the pulmonary artery due to enlarged lymph nodes might also benefit from immunosuppressive treatment [10].

#### Oxygen therapy

Hypoxic vasoconstriction due to decreased arterial oxygen pressure is one of the possible mechanisms for PH. Oxygen therapy is recommended in a subgroup of patients. Oxygen administration was shown to reduce pulmonary vascular resistance in patients with pulmonary arterial hypertension; however, there are no randomised data for long-term oxygen therapy [2]. Based on a study in patients with chronic obstructive pulmonary disease, the European guidelines for PH recommend the use of oxygen in patients with a partial arterial blood oxygen pressure < 8.0 kPa [2].

### PH-targeted therapy

PH-targeted therapy is reserved for group 1 and 4 PH patients only. Over the last years, treatment of SAPH using the PH-targeted therapy carefully shows some improvement in selected patients, especially in the presence of specific vasculopathy or increased vasoreactivity. This is based on case series only. Due to the lack of randomised controlled clinical trials and potential severe adverse events of treatment, the use of PH-targeted therapy in sarcoidosis is currently off-label. It should be noted that PH-targeted therapy can be ineffective and sometimes even dangerous for patients with fibrotic remodelling. These patients are less likely to respond to vasodilators, and treatment might worsen oxygenation, since the physiological hypoxaemic vasoconstriction is inhibited. As a result, there is an increased blood flow to areas with poor ventilation that might lead to a shunt or ventilation/perfusion discrepancy, as described in patients with idiopathic pulmonary fibrosis [43]. Therefore, significant hypoxia can be a contraindication for PH-targeted treatment. It may also cause acute pulmonary oedema and hypoxaemia through preferential vasodilation of the pulmonary arteries, if there is a down-stream venular obliteration by granulomas [16]. Therefore, treating physicians should be very careful before initiating these drugs in sarcoidosis patients, and treatment decisions should only be made on an individual basis in an expert centre by a multidisciplinary team, based on a goal-oriented approach with predefined targets.

Below, the PH-targeted therapies in SAPH will be discussed. Outcomes of the most important studies are summarised in Tab. 4.

**Tab. 4:** Overview of literature on pulmonary hypertension targeted treatment in sarcoidosis

Author	Year and type	Treatment	*N*	mPAP	PVR	CO	Other outcomes and adverse events
*Prostacyclin analogues*							
Preston [15]	2001 CS	Nitric oxide	8	↓ 18 %	↓ 31 %	↑ 12 %	
Preston [15]	2001 CS	Epoprostenol	6	↔	↓ 25 %	↑ 25 %	
Fisher [16]	2006 CS	Epoprostenol	7	↓ 21 %	↓ 45 %	↑ 44 %	↑ NYHA 1–2 classes Decreased Pao2 in 3/7 One death
Baughman [45]	2009 CS	Iloprost	15	↓ 15 %	↓ 45 %	–	↑ 12 % 6MWT ↑ QOL Decrease in Pao2 in 2/15
*Endothelin receptor antagonists*							
Judson [46]	2011 CS	Ambrisentan	21	–	–	–	↔ 6MWT, NYHA, QOL 38 % discontinued due to ↑ oedema and dyspnoea
Baughman [26]	2014 RCT	Bosentan	35	↓ 11 %	↓ 28 %	–	↔ 6MWT
*Phosphodiesterase 5 inhibitors*							
Milman [9]	2008 CS	Sildenafil	12	↓ 19 %	↓ 48 %	↑ 36 %	↔ 6MWT
*Combined studies*							
Barnett [25]	2009 CS	Sildenafil, bosentan, epoprostenol	22	↓ 20 %	↓ 39 %	↔	↑ 6MWT
Dobarro [47]	2013 CS	Bosentan (2) or sildenafil (9)	11	↔	↔	↔	↑ 6MWT ↔ NYHA

*6MWT* six-minute walk test, *CO* cardiac output, *CS* case series, *mPAP* mean pulmonary artery pressure,* NYHA* New York Heart Association*, PaO2* partial arterial oxygen pressure,* PVR* pulmonary vascular resistance, *QoL* quality of life*, RCT* randomised controlled trial

#### Prostacyclin analogues

During the 1980s, prostanoids were the first agents used in an attempt to treat PH in sarcoidosis [44]. Decades later, a case series appeared evaluating epoprostenol for moderate to severe PH in eight sarcoidosis patients [16]. Most patients responded. One patient, with pulmonary veno-occlusive disease as an underlying mechanism of PH, developed congestive heart failure leading to pulmonary oedema. In a study of 15 patients with inhaled iloprost [45], six had improved haemodynamics and significant improvement in quality of life. As a clinical outcome, only three patients had more than 30 m improvement in their 6MWT.

#### Endothelin receptor antagonists

Endothelin receptor antagonists have been more extensively investigated, particularly bosentan. One double-blind, placebo-controlled trial investigated 35 PH patients with all different sarcoidosis stages, presenting with New York Heart Association class II or III symptoms, low PAWP and stable on immunosuppressive therapy for at least three months [26]. Bosentan improved the mean PAP and pulmonary vascular resistance. However, this 16-week period of treatment did not significantly improve the 6MWT or quality of life. A prospective case series of 21 sarcoidosis patients of all stages studied ambrisentan, another endothelin receptor antagonist. This resulted in preliminary cessation of treatment in > 50 % of the patients [46], mainly due to oedema and/or dyspnoea. The ten patients who completed the study seemed to have an improved quality of life. However, the study was not powered to have significant results. There are no data regarding the outcomes of macitentan for treating PH in sarcoidosis.

#### Phosphodiesterase 5 inhibitors

Milman and colleagues [9] reported improvement in the haemodynamics in patients with PH awaiting lung transplant, treated with sildenafil. There was no change in the 6MWT distance. Some cases suggest phosphodiesterase 5 inhibitors to be more successful in treating sarcoidosis patients with less severe PH [25, 47].

### Lung transplant

For patients with end-stage pulmonary parenchymal sarcoidosis or SAPH who failed therapy, single or bilateral lung transplant can be a feasible option. Between January 1995 and June 2012, 954 sarcoidosis patients out of 18 countries underwent lung transplant [48]. Median survival for sarcoidosis patients after lung transplant is calculated to be approximately 8.5 years [48].

## Conclusion

Sarcoidosis patients have an increased risk of developing PH. The exact prevalence of PH in sarcoidosis is unknown. To date, identifying sarcoidosis patients at risk for PH is still a major challenge. There is no validated algorithm to refer patients for PH analysis, which might lead to under-detection of PH. Moreover, echocardiography has limited diagnostic accuracy, especially in patients with parenchymal involvement. More extensive diagnostic tests and evaluation of treatment possibilities should therefore be considered in specialised centres with a multidisciplinary expert team. Treatment of PH in sarcoidosis is challenging, since there is a lack of robust data. Case series evaluating treatment options involving PH-targeted therapy are arising for a subgroup of patients. Therefore, further research aiming to optimise the diagnostics and treatment for PH in pulmonary sarcoidosis is warranted.
